# Supplementary material for: Narrow transmission bottlenecks and limited within-host viral diversity during a SARS-CoV-2 outbreak on a fishing boat
Source: bioRxiv. 2022 Feb 9:2022.02.09.479546. Preprint. [Version 1] doi: 10.1101/2022.02.09.479546 (PMC8845427; doi:10.1101/2022.02.09.479546)
Supplement: 1 [file NIHPP2022.02.09.479546V1-supplement-1.pdf]

bioRxiv preprint doi: https://doi.org/10.1101/2022.02.09.479546; this version posted February 9, 2022. The copyright holder for this preprint (which was not certified by peer review) is the author/funder, who has granted bioRxiv a license to display the preprint in perpetuity. It is made available under aCC-BY 4.0 International license.

Supplement

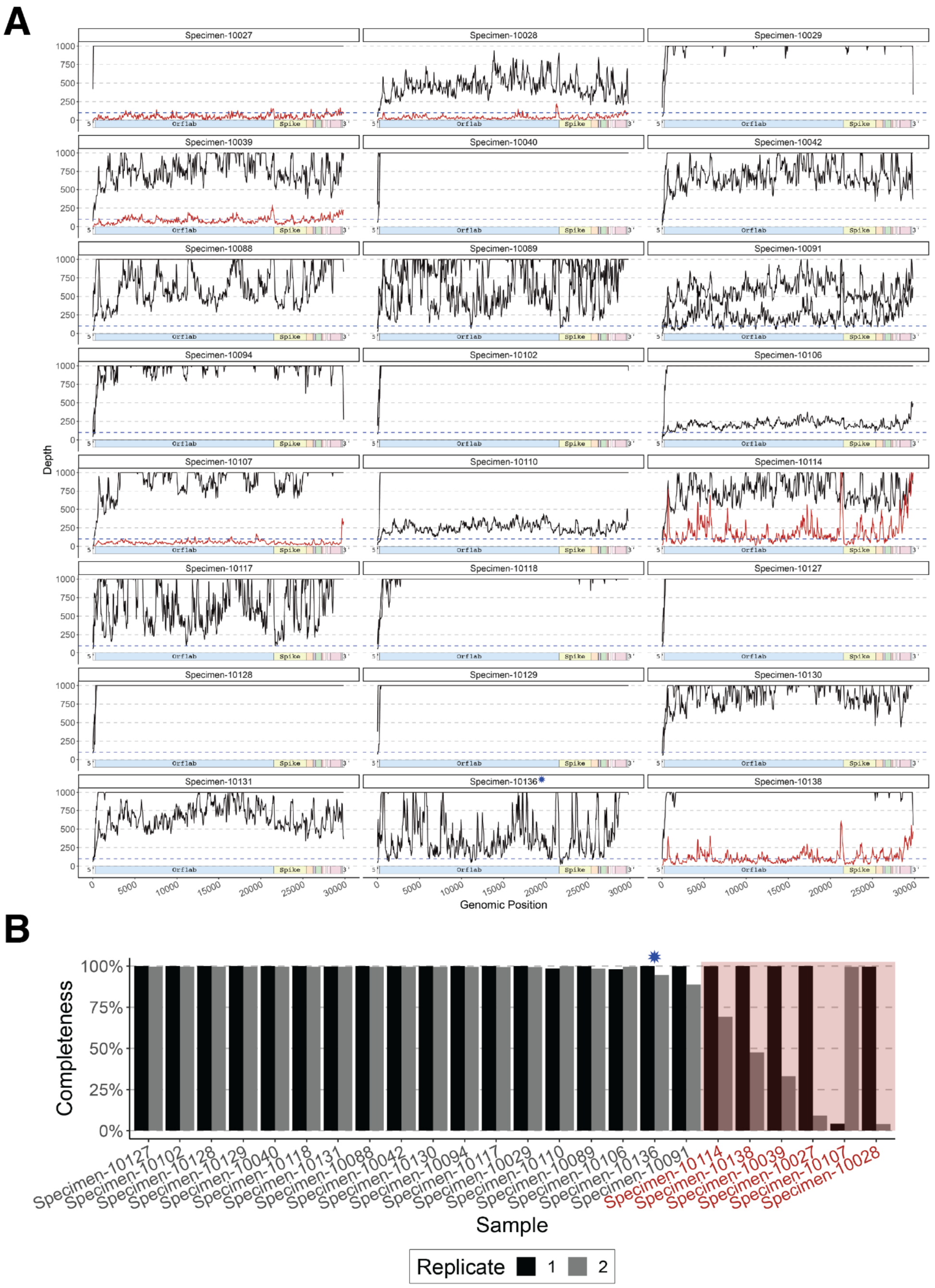

Supplementary Figure 1. Samples were filtered by completeness. (A) The pattern of sequencing depth for each replicate of all 23 specimens from the boat, and one control sample that was not from the boat (Specimen-10136, labeled with a blue asterisk), that we resequenced for this study. The number of reads per site is capped at 1000X coverage. Samples colored in red have less than 80% of the genome covered by 100X reads. (B) Completeness refers to the percentage of the genome covered by more than 100X reads. Samples colored and highlighted in red have at least one replicate with less than 80% of the genome covered by 100X reads. These samples were excluded from the downstream variant analysis.

bioRxiv preprint doi: https://doi.org/10.1101/2022.02.09.479546; this version posted February 9, 2022. The copyright holder for this preprint (which was not certified by peer review) is the author/funder, who has granted bioRxiv a license to display the preprint in perpetuity. It is made available under aCC-BY 4.0 International license.

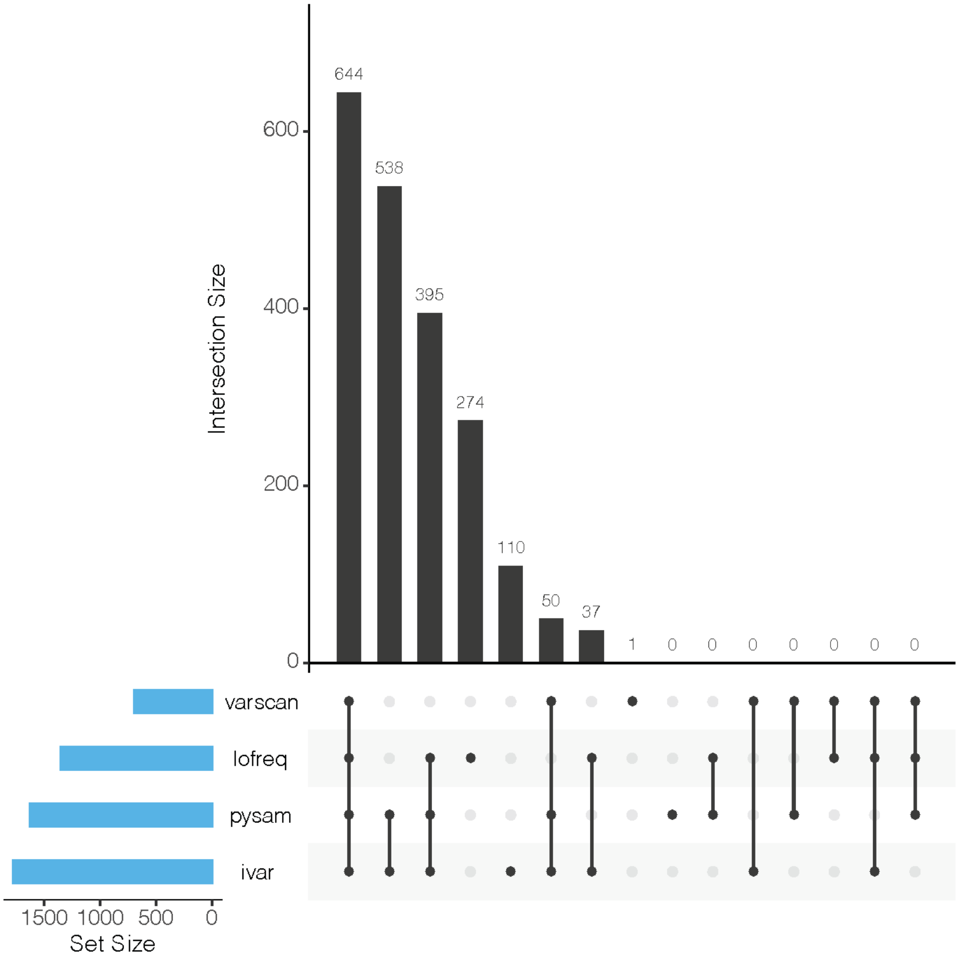

Supplementary Figure 2. Comparison between different variant calling methods. An UpSet plot shows the overlap in the sets of SNPs called by three different variant calling methods – varscan2, lofreq, ivar, and our custom python script using pysam (*Citations*). Variants were covered by more than 100X reads and present at greater than 2% frequency to be included in the set for each variant caller. The majority of variants are called by all four methods. No variants are called by our custom script that aren't identified by at least one other method.

bioRxiv preprint doi: https://doi.org/10.1101/2022.02.09.479546; this version posted February 9, 2022. The copyright holder for this preprint (which was not certified by peer review) is the author/funder, who has granted bioRxiv a license to display the preprint in perpetuity. It is made available under aCC-BY 4.0 International license.

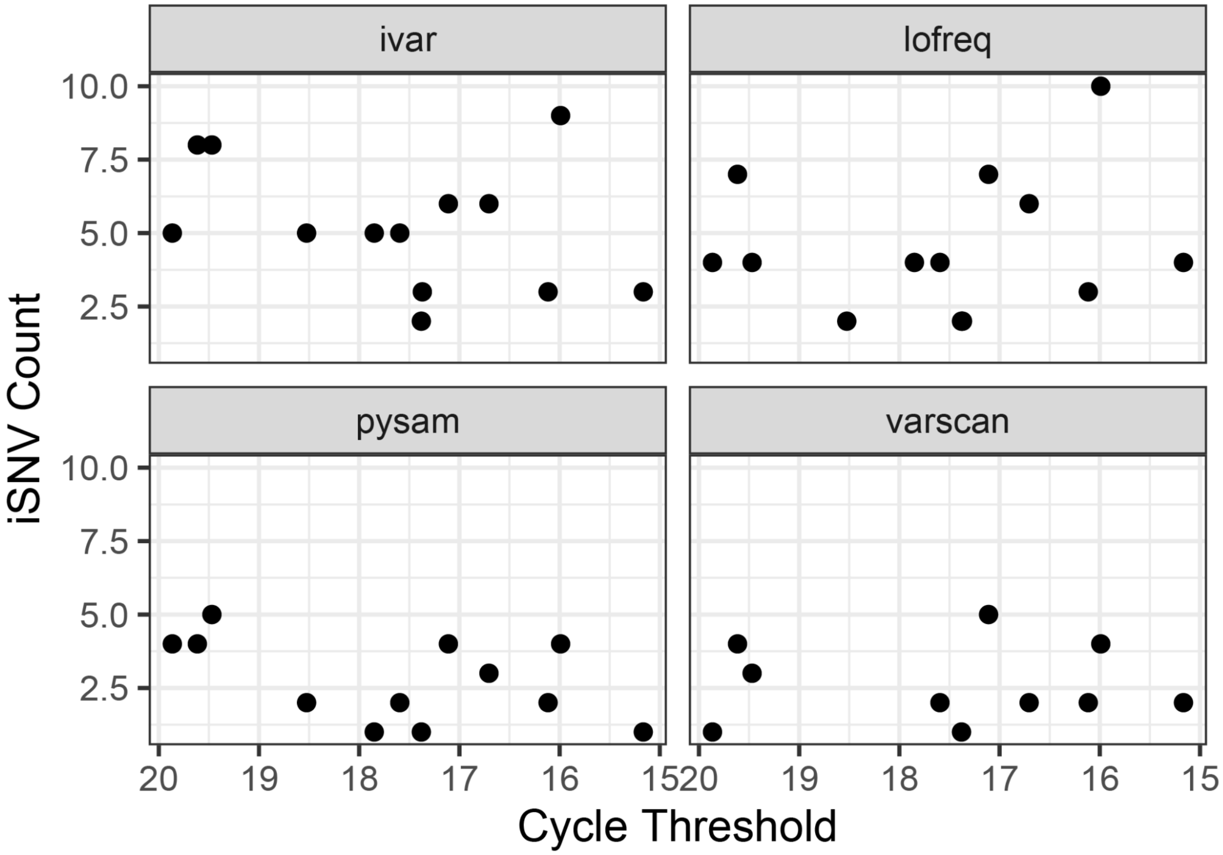

Supplementary Figure 3. Ct value does not correlate with the number of polymorphisms. Regardless of the variant calling method used, the Ct value of the original nasal swab does not correlate with the number of variants called after filtering out low-frequency (>2%) and poorly covered (>100X) variants. Only samples that passed our quality controls for sequencing completeness (Fig. S3B) and concordance (Fig. 2) were included in this analysis.

bioRxiv preprint doi: https://doi.org/10.1101/2022.02.09.479546; this version posted February 9, 2022. The copyright holder for this preprint (which was not certified by peer review) is the author/funder, who has granted bioRxiv a license to display the preprint in perpetuity. It is made available under aCC-BY 4.0 International license.

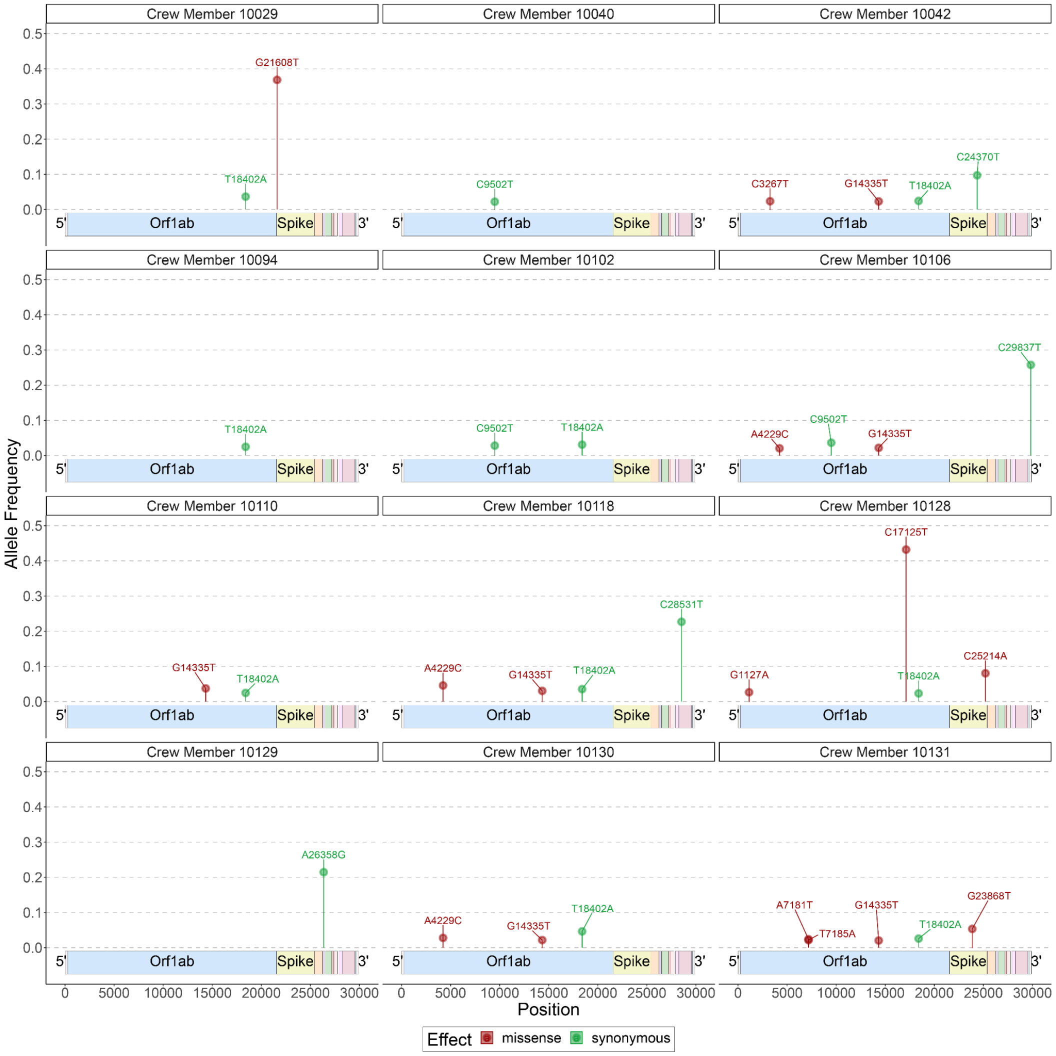

Supplementary Figure 4. There is no discernable pattern of minor variants in the genome. Plot showing every minor variant (>50% allele frequency) identified across the crew members that passed our quality filters. We included variants if they were present in more than 2% of greater than 100 reads.

bioRxiv preprint doi: https://doi.org/10.1101/2022.02.09.479546; this version posted February 9, 2022. The copyright holder for this preprint (which was not certified by peer review) is the author/funder, who has granted bioRxiv a license to display the preprint in perpetuity. It is made available under aCC-BY 4.0 International license.

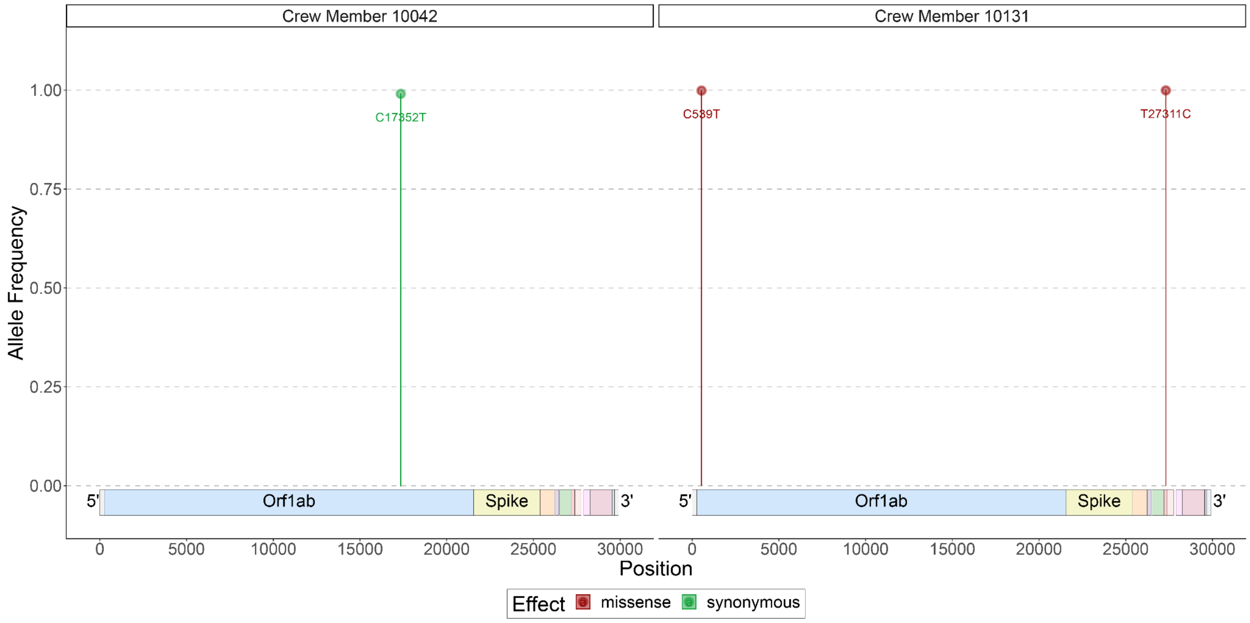

Supplementary Figure 5. Distribution of fixed mutations in the genome. Plot showing fixed variants identified across the crew members that passed our quality controls. We included variants if they were present in 98% or more of at least 100 reads. Mutations that are present in the 5' and 3' UTRs are excluded from this plot.

bioRxiv preprint doi: https://doi.org/10.1101/2022.02.09.479546; this version posted February 9, 2022. The copyright holder for this preprint (which was not certified by peer review) is the author/funder, who has granted bioRxiv a license to display the preprint in perpetuity. It is made available under aCC-BY 4.0 International license.

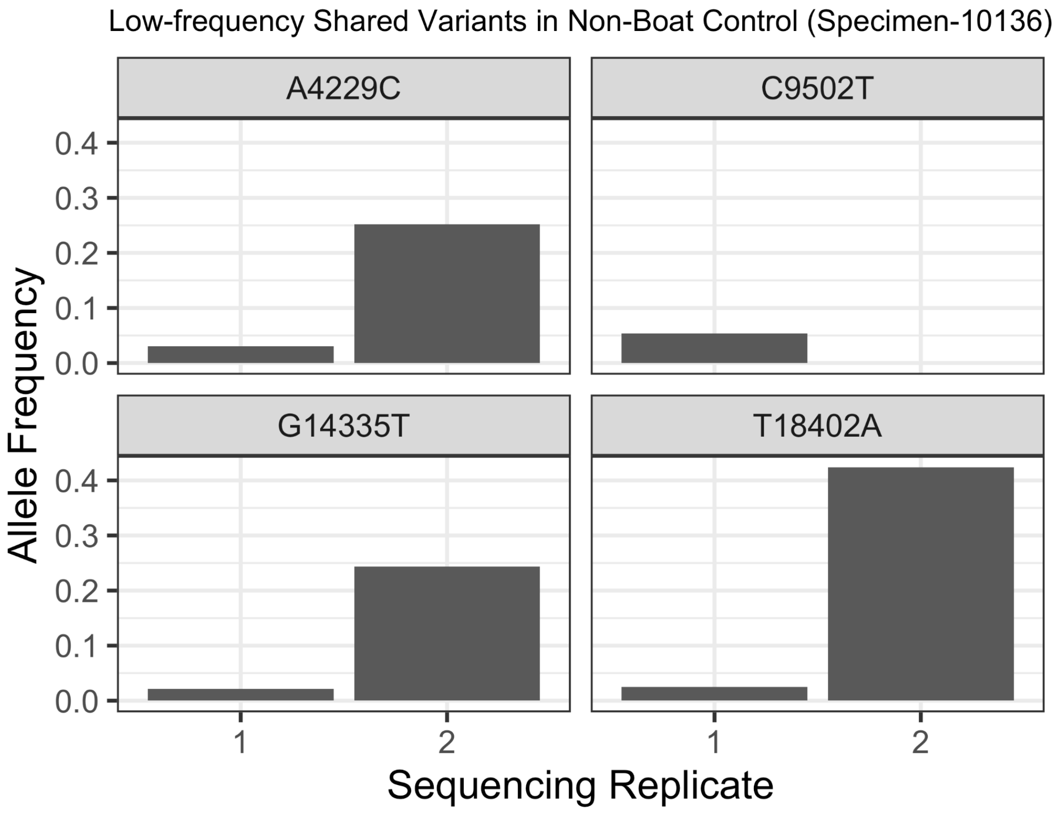

Supplementary Figure 6. Low frequency shared variants are present in the non-boat control specimen. Four variants shared at low-frequency between crew members are also detected in a specimen not collected from the boat but included as a control in both sequencing runs (Specimen 10136). This observation suggests that these are not de novo low-frequency variants that arise on the boat and spread between the crew, but rather sequencing contamination or variant calling errors common to samples from the two sequencing runs.
